# Gaze behavior in response to affect during natural social interactions

**DOI:** 10.3389/fpsyg.2024.1433483

**Published:** 2024-10-14

**Authors:** Antonia Vehlen, Artem V. Belopolsky, Gregor Domes

**Affiliations:** ^1^Department of Biological and Clinical Psychology, University of Trier, Trier, Germany; ^2^Department of Movement Sciences, Vrije Universiteit Amsterdam, Amsterdam, Netherlands; ^3^Institute for Cognitive and Affective Neuroscience, University of Trier, Trier, Germany

**Keywords:** gaze behavior, social interaction, facial affect, dynamic analysis, eye-tracking

## Abstract

Attention in social interactions is directed by social cues such as the face or eye region of an interaction partner. Several factors that influence these attentional biases have been identified in the past. However, most findings are based on paradigms with static stimuli and no interaction potential. Therefore, the current study investigated the influence of one of these factors, namely facial affect in natural social interactions using an evaluated eye-tracking setup. In a sample of 35 female participants, we examined how individuals' gaze behavior responds to changes in the facial affect of an interaction partner trained in affect modulation. Our goal was to analyze the effects on attention to facial features and to investigate their temporal dynamics in a natural social interaction. The study results, obtained from both aggregated and dynamic analyses, indicate that facial affect has only subtle influences on gaze behavior during social interactions. In a sample with high measurement precision, these findings highlight the difficulties of capturing the subtleties of social attention in more naturalistic settings. The methodology used in this study serves as a foundation for future research on social attention differences in more ecologically valid scenarios.

## 1 Introduction

Interactions are a major part of human social life. Success relies on allocating attention to key social cues (i.e., diagnostic cues). In social interactions cues become diagnostic, which allow conclusions to be drawn about the identity of the interaction partner, his or her feelings, the direction of attention, and the content of what is being said. The face, and in particular the eye region of the interaction partner, are a rich source of social information and therefore attract the most attention (Birmingham et al., 2009; Devue et al., 2012; Yarbus, 1967). Eye movements are closely tied to the cognitive and emotional processes underlying social interactions, as proposed by the *eye-mind hypothesis*, which suggests a direct link between where we look and what we process cognitively (Just and Carpenter, 1984). Retrieving social information is facilitated when the partner responds with direct gaze (Senju and Johnson, 2009). Apart from information retrieval, eye contact has the function of conveying information to the interaction partner, e.g., about one's own current mental state or intended further actions (*dual function of gaze*; Argyle and Cook, 1976; Risko et al., 2016). Despite a general preference for faces and the eye region, studies reveal substantial variance in social attention.

The recording of gaze behavior in standardized setups was used as an indicator of attention allocation (Wright and Ward, 2008), and systematic experimental manipulations helped identify factors contributing to the variance in attentional preferences during face viewing (for review see: Dalmaso et al., 2020; Hadders-Algra, 2022). On the observer side, factors such as age, cultural background, and potential psychopathologies have been shown to influence these processes. While attention to faces in general seems to decrease with age (De Lillo et al., 2021), individuals from Western cultures have been found to fixate more on the mouth region compared to individuals from Asian cultures (Senju et al., 2013). Furthermore, altered processing of the eye region has been linked to several pathologies such as schizophrenia (Loughland et al., 2002), autism spectrum disorder (Itier and Batty, 2009; Setien-Ramos et al., 2022), and social anxiety (Chen et al., 2020). It is believed, that altered attentional processing contribute to impaired social functioning in these disorders.

In addition to observer characteristics, variance in attentional preferences can also be explained by factors inherent to the stimulus (e.g., interaction partner). One factor that has been studied continuously is the affective state of the stimulus. Affective states, such as those mediated by emotional expressions, have been linked to attentional preferences, as, for example, the mouth region attracts more attention when positive affect, such as a happy stimulus, is viewed (Beaudry et al., 2014; Calvo et al., 2018; Green et al., 2003). Findings are less clear for negative stimuli and can vary depending on factors such as affect type and study design. Some studies have reported an increase in attention to the eye region for sad and angry stimuli (Beaudry et al., 2014; Calvo et al., 2018; Eisenbarth and Alpers, 2011), while others have observed a decrease in attention for fearful and angry stimuli (Hunnius et al., 2011). These heterogenous findings indicate the complexity and variability of the relationship between affect and gaze behavior.

Although these studies have provided valuable insights into information retrieval through gaze behavior, the standardization used limited the setups to computer screens and thus never captured the signaling function of gaze. The impact of this simplification became clear through a series of studies applying a *waiting room paradigm* (Grossman et al., 2019; Horn et al., 2022; Kulke et al., 2023; Laidlaw et al., 2011; Rösler et al., 2021). In these studies, it was demonstrated that attention to faces varied significantly depending on the social context (e.g. screen vs. live). With advancing technology, the field has developed more naturalistic setups that capture the dynamics of gaze behavior, allowing for greater complexity in the study of social attention (Hessels et al., 2017; Tönsing et al., 2022). However, such naturalistic studies are very challenging to conduct, as they require extensive knowledge of the factors that influence data quality and the possibilities of standardization and validation (Nebe et al., 2023; Vehlen et al., 2021, 2022). In addition to the technical challenges, data collection in these naturalistic studies is often so complex that it requires extensive training for the individuals involved. In order to ensure that observed behavior in social interactions is not influenced by personal factors, experimenters and research assistants must learn to modulate their behavior as consistently and convincingly as possible. Despite these challenges, recent reviews emphasize the importance of integrating the historical best practices of eye-tracking with these modern methodologies, advancing the understanding of gaze functions in complex and interactive environments (Carter and Luke, 2020; Hessels, 2020).

The present study applied such a setup to investigate gaze behavior in natural social interactions, as evaluated by Vehlen et al. (2021). The setup was used to observe gaze dynamics in response to facial affect modulations by an interaction partner. More specifically, participants interacted with trained research assistants (RAs) who were instructed to show brief sequences of positive and negative facial affect at defined time points during a structured conversation, while participants' gaze behavior was recorded using remote eye-tracking. Based on the above literature of standardized setups, we hypothesize that there will be significant differences in the distribution of attention to the eye and mouth regions depending on the observed facial affect. Specifically, we expect that positive facial expressions will result in greater attention to the mouth region, negative expressions will lead to greater attention to the eye region. Furthermore, we hypothesize that these attentional patterns will exhibit time-dependent variability, reflecting the dynamics of natural social interactions. Specifically, we predict that differences in attention distribution will be most pronounced during the affect modulation period, rather than before or after. To capture these nuances, we have employed analytical methods that allow us to observe and quantify these dynamic effects over time. The longer-term goal is to contribute to our understanding of successful interactions, with potential applications in fields such as psychotherapy or robotics (Hessels, 2020).

## 2 Methods

### 2.1 Participants

Fifty-one female participants took part in the study. They were recruited via internal communications of the University of Trier and via flyers distributed on campus. Exclusion criteria consisted of visual impairments exceeding ± 1.5 diopters, any type of prescriptive visual aid other than soft contact lenses, acute eye inflammation and prior eye surgery. In the non-clinical sample psychotherapeutic treatment in the last 2 years, neurological diseases, and residual symptoms of a brain injury or the intake of psychotropic drugs also led to exclusion of participants. The sample was limited to heterosexual female participants to reduce attraction effects in interactions with female RAs. Six participants were excluded from analysis because of technical problems with the eye-tracker during testing sessions (*n* = 6). Four additional participants were excluded because the facial affect modulation was not performed, as reported by the research assistant who did not receive signals through the headphones (*n* = 4). Three participants were excluded because they commented on the affect modulation performed by the RAs (*n* = 3). During the session, other participants retrospectively reported watery eyes (*n* = 1), visual impairment above the predefined threshold (*n* = 1) and astigmatism (*n* = 1), which also led to exclusion. The final sample consisted of 35 women with a mean age of 22.3 ± 3.1 years (range: 18–29 years).

The study was conducted in accordance to the Declaration of Helsinki and was approved by the ethics committee of the University of Trier. All participants were informed about the study, gave written informed consent, and received compensation of €10. The pictures on which a female person is depicted are used with the permission of this person, who has given her informed consent for publication.

### 2.2. Setup, calibration and validation

Gaze data was recorded at a sampling frequency of 120 Hz with a Tobii X3-120 remote infrared eye-tracker and the Tobii Studio Pro software (version 3.4.5). The eye-tracker was placed on a square table using a fixed monopod on which it was mounted at a 28° angle. The scene was recorded with a webcam (Logitech c920; 30 fps, 1,920 × 1,080 px resolution) attached above the heads of the participants. The operating distance (distance between the participant's eyes and the eye-tracker) was set at 65 cm, while the viewing distance (distance between the eyes of the interaction partners) was constant at 131 cm (detailed description and an illustration of the setup with the exact distances can be found here: Vehlen et al., 2021). The nine-point calibration sheet was attached to a partition wall and covered an area of 40 × 40 cm (21.6 × 21.6° visual angle) with a inter-point distance of 20 cm (~8.7° visual angle). The sheet was placed approximately at the height at which the face of the RA was expected to be. The calibration was followed by three validation sequences over the course of the entire testing session. The first validation sequence immediately followed the calibration by repeating five of the calibration points (except for the points in the four corners) in random order (*wall validation*). Another validation sequence took place just before the interaction by instructing the participant to fixate facial features of the interaction partner (left eye, right eye, nose, and mouth; *pre-validation*). Since the facial features are moving target points, a frame-wise definition of the corresponding coordinates had to be performed. This was possible with the facial recognition software OpenFace (Amos et al., 2016), from whose facial landmarks the target points were calculated. The interaction was followed by another validation sequence on the face of the interaction partner (*post-validation*). Data quality was also estimated during the interaction and correlated with the movement of the interaction partner. These results can be considered in more detail in the evaluation study of the setup (Vehlen et al., 2021) and are not included in this paper.

### 2.3. Face-to-face interaction

**Figure 1:**
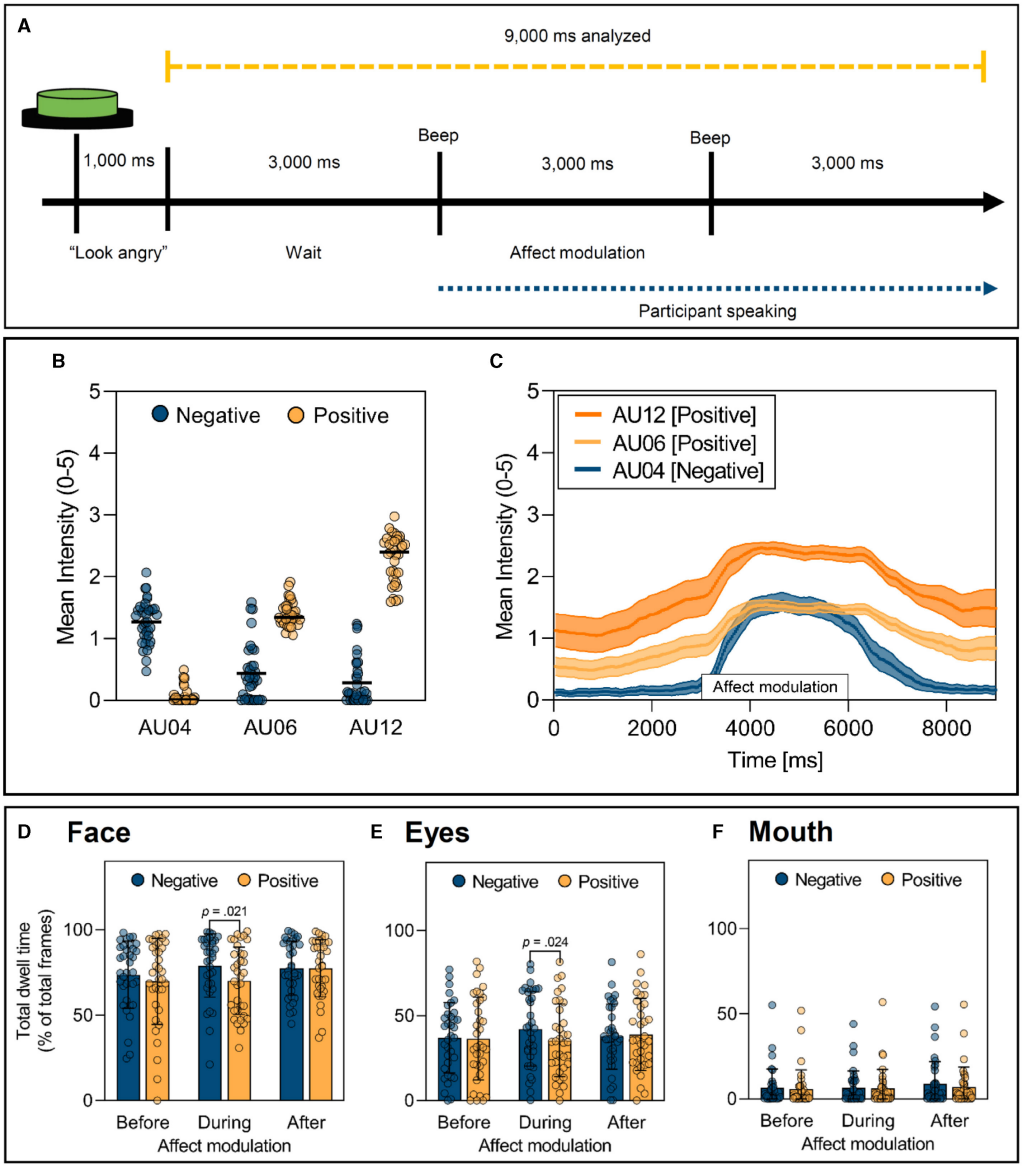
Procedure and effect of facial affect modulation within natural social interactions. **(A)** Timing of affect modulation. Instructions (e.g., “Look angry”) are transmitted through in-ear headphones worn by the Research Assistant (RA). The blue line indicates the time during which the participant speaks. The yellow line illustrates the data analyzed in the study. **(B, C)** Activation of Action Units (AUs) during affect modulation. **(B)** Mean intensity of AU activation during the three seconds of positive and negative affect averaged over all trials. Black lines represent the mean intensities over all participants. **(C)** Mean intensity of AU04, AU06 and AU12 activation over time (9 seconds) averaged over all trials and participants. Solid lines represent mean values with corresponding 95%-confidence intervals visible by the shaded areas. **(D–F)** Gaze on facial Areas Of Interest (AOIs) as a function of affect type and time. **(D)** Summed total dwell times on all facial AOIs. **(E)** Total dwell times on the eyes. **(F)** Total dwell times on the mouth. Before = 0–3,000 ms; During = 3,000–6,000 ms; After = 6,000–9,000 ms. Bars represent *M* ± SD.

For the face-to-face interaction, two RAs were trained to modulate their facial affect in response to instructions transmitted through in-ear headphones. They were taught to modulate their facial expressions to express negative, neutral, and positive affect. During the actual interactions the background (gray curtains) and the clothing of the RAs (dark without prints) were kept constant. The interaction sequences were structured by 12 questions from a published paradigm (Aron et al., 1997). The questions were alternately read aloud by the RA and the participant. Both the RA and the participant answered each question, resulting in two responses for each question. The questions were designed to facilitate interpersonal connection and encompassed a diverse array of topics. Examples included selecting a dinner guest from any individual in the world, discussing aspirations for fame, and reflecting on personal habits and life narratives. The full list of questions can be found in the Supplementary material. To ensure consistency and promote a positive interaction atmosphere, the RAs prepared their responses in advance. Prior to providing their answers, both the RA and the participant would press a movable buzzer on the table. When the participant pressed the buzzer and answered the question, the buzzer triggered a Raspberry Pi running an internal script that sent the instructions (e.g., “Look angry”) to the RAs' in-ear headphones (for details see Figure 1A). The whole interaction lasted on average 17.31 ± 3.36 min. The RAs showed each affect types (negative, neutral, and positive) four times during the interaction while participants were responding to the questions. The order of affect types was randomized.

### 2.4. Procedure

After an online screening that assessed the exclusion criteria, participants were invited to the laboratory. Each testing session began with the calibration of the RA, who was presented to the participants as an unknown interaction partner. However, in this study, only the participants' gaze data were analyzed. Subsequently, the participant was calibrated. Each calibration was followed by the first validation sequence. When the calibration wall was replaced with the RA's chair, both interaction partners, now seated across from each other, performed another validation sequence on the face of the interaction partner. The social interaction then took place, followed by another round of face validation. Last, participants completed questionnaires that captured constructs such as social anxiety, gaze anxiety and autistic traits. Summary statistics of these measures are provided in Supplementary Table S1.

### 2.5. Data analysis

#### 2.5.1. Preprocessing

All calculations of gaze data were based on averaged binocular data and the total dwell times were chosen as gaze measure. In total dwell times all gaze behavior (fixations, saccades, etc.) directed at a given region within a given time period are analyzed.

#### 2.5.2. Robustness, precision, accuracy

Robustness or trackability is a measure of the amount of data lost due to failed pupil detection, blinks of the participant or looks outside the tracking area. Lower values correspond to higher data loss. The accuracy can be understood as a measure of the validity of the gaze data and is high if the gaze coordinates recorded by the eye-tracker match the target point of the observer. Thus, low values correspond to high accuracy. Precision reflects the reliability of the gaze data and is high when continuous gaze at a given point in space results in gaze coordinates with a low dispersion. Again, low values correspond to high precision. More detailed information on the calculation of these three measures can be found here (Vehlen et al., 2021).

#### 2.5.3. Validation of facial affect modulation

To observe the effect of facial affect on gaze behavior, facial affect had to be accurately produced by the RAs. To ensure the success of this modulation, video sequences of the 3 s of facial affect modulation were analyzed with the Facial Action Coding System (FACS; Ekman and Friesen, 1978) implemented in OpenFace (Amos et al., 2016). FACS is a comprehensive, anatomically based system for describing observable facial movements. It identifies specific facial muscle movements, known as Action Units (AUs), which correspond to particular emotions. The analysis focused on the mean intensities of three specific AUs:

- AU04 (Brow Lowerer): this action unit involves the lowering of the eyebrows, typically associated with negative emotions such as anger or sadness.
- AU06 (Cheek Raiser): commonly involved in the expression of happiness, contributing to the formation of crow's feet around the eyes.
- AU12 (Lip Corner Puller): also associated with happiness, resulting in the upward movement of the lip corners.

It was expected that positive affect would be dominated by activation of AU06 and AU12, whereas negative affect would be dominated by activation of AU04. In addition, the average time course of AU04 and AU06 activation over all participants was visualized to check for temporal deviations in affect modulation by the RAs. Thereby it was expected that activation of AUs would be highest during affect modulation and that there should be no pronounced temporal fluctuations in the onset of activation. After analysis of AUs, three trials of negative affect (2.21%) had to be excluded from the eye-tracking data analysis because the corresponding AUs were not activated.

#### 2.5.4 Definition of areas of interest

**Figure 2:**
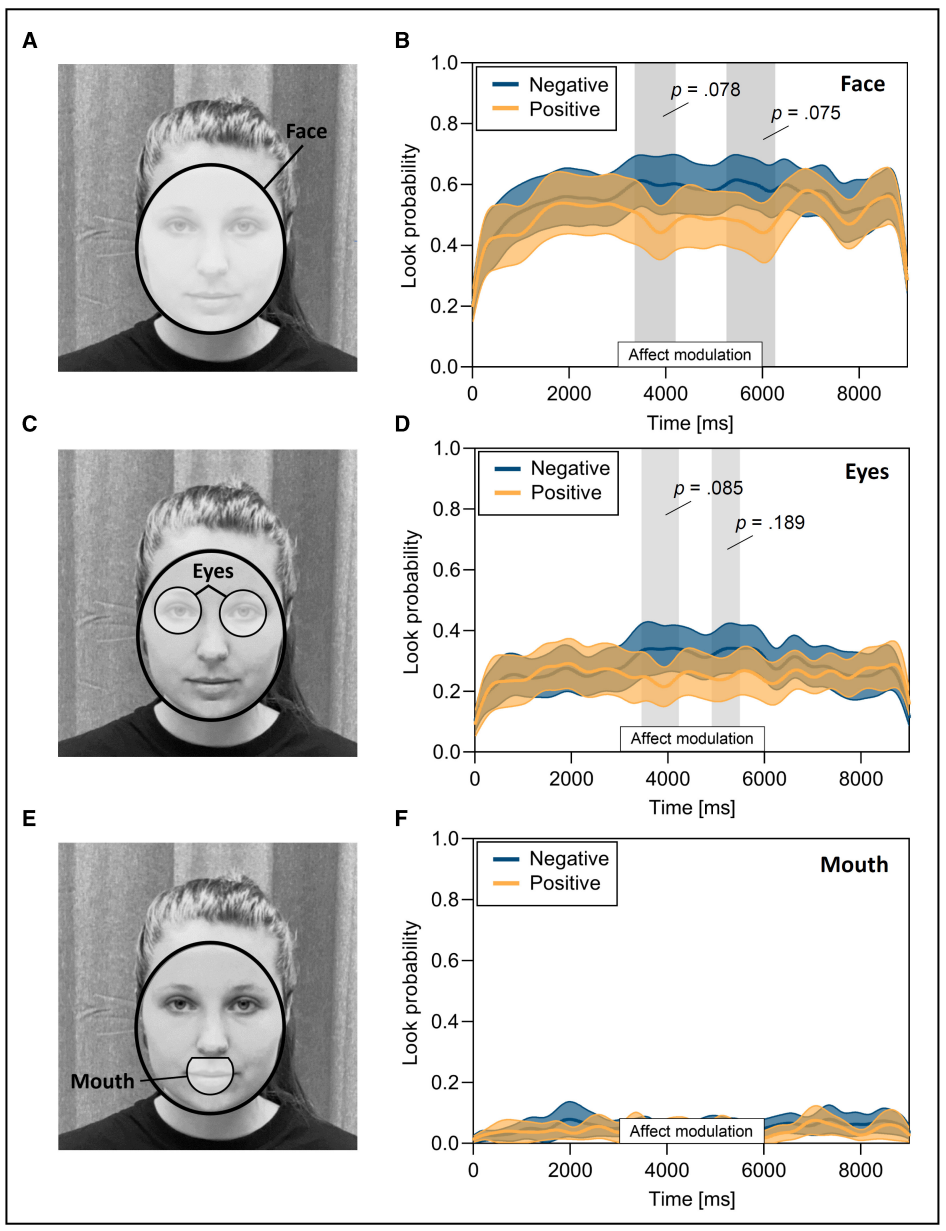
Look probability for facial areas of interest (AOIs) in response to positive and negative affect. **(A)** An ellipse around the face generated through landmark detection (AOI face) (Amos et al., 2016). **(B)** Look probability over time on the AOI face in response to facial affect modulation. **(C)** AOI eyes automatically generated with the Limited-Radius Voronoi Tessellation method (LRVT; Hessels et al., 2016) and a radius of 1° visual angle. **(D)** Look probability over time on the AOI eyes in response to facial affect modulation. **(E)** Automatically generated AOI mouth. **(F)** Look probability over time on the AOI mouth in response to facial affect modulation. Vertical gray shaded areas mark differences between conditions. Solid lines represent the mean look probability with the corresponding 95% confidence intervals. Written informed consent was obtained from the individual for the publication of the identifiable images.

All analyses of gaze data required the definition of areas of interest (AOIs) on the face of the RA to make assumption about the participant's gaze direction. We thus applied the facial recognition software OpenFace (Amos et al., 2016) and used the *Limited-Radius Voronoi Tessellation method* (LRVT; Hessels et al., 2016) to automatically generate these AOIs. Based on the data quality obtained in the study, we chose a radius of 1° visual angle (Vehlen et al., 2022) (see Figures 2C, E). We also added an ellipse around the face as an additional AOI (see Figure 2A). All available gaze data that were not directed to one of the facial AOIs were classified as AOI surrounding. Gaze data not available in the dataset were classified as missing. Missing data could be due to the participant looking outside the tracking area, blinking, or the eye-tracker losing the participant's eyes.

### 2.6 Statistical analyses

For each participant, all statistical analyses were based on eight trials of facial affect modulation (4× positive and 4× negative) within the social interaction, each lasting 9 s. Three seconds before the facial affect modulation, 3 s during and 3 s after (see Figure 1A). The length of the last segment was based on the minimum length of participants' responses to the questions in the social interaction.

We first analyzed gaze data based on aggregation using repeated-measures ANOVA. AOIs (eyes, nose, mouth, rest of face, surrounding), time (0–3,000 ms, 3,000–6,000 ms and 6,000–9,000 ms) and affect type (negative and positive) served as within-subject factors. In a second step, the AOI face (sum of AOI eyes, nose, mouth and rest of face) and the AOIs eyes and mouth were analyzed separately by means of repeated-measures ANOVA with time and affect type as within-subject factors. These analyses were conducted in R using the *afex* package (Singmann et al., 2016) with the level of significance set to *p* < 0.05. In case the assumption of sphericity was violated, Greenhouse-Geisser correction was applied. Effect sizes are reported as partial eta squared ($\eta_{\text{p}}^{2}$). Planned comparisons with Bonferroni corrections were used to test whether differences in total dwell times on the face, eyes, and mouth actually occurred as a response to the affect modulation. $\eta_{\text{p}}^{2}$ values were estimated from the *t*-ratios as a measure of effect size.

The analyses were repeated with a focus of underlying dynamics in gaze data. For that, the total dwell times served as the basis for applying the *Smoothed Proportion of Looks Over Time* (SPLOT; Belopolsky, 2020) method. This method was developed as an alternative to classical AOI-based analysis, where temporal information is usually lost due to aggregation. The issue of multiple testing in these type of analyses is avoided by the application of cluster based permutation testing (Maris and Oostenveld, 2007; van Leeuwen et al., 2019). We implemented the method in Matlab (2018, version 9.4.0) and used it specifically to compare look probability between trials with negative and positive affect for the AOI face as well as the AOI eyes and mouth. For each comparison, we computed 1,000 iterations of permutation. During each iteration, the labels for the negative and positive affect trials were randomly permuted to break any association between the affect conditions and the observed gaze data. We then calculated the paired *t*-statistic for each permuted data set. A cluster was defined when two consecutive time points reached significance. We then used the summed *t*-statistics of the largest cluster to generate a null distribution of the test statistics. After creating the null distribution, we set a threshold for the clusters based on the 95th percentile of the distribution to identify significant differences. We than compared the two affect conditions by means of paired *t*-tests and again defined clusters. These clusters were considered significant only when the summed *t*-statistics were above the defined threshold derived from the distribution. The degree of smoothing was set to a standard deviation of 100 ms to correspond to the minimum duration of fixations (Belopolsky, 2020). We chose to use the average Cohen's *d* for each cluster as a measure of effect size (Meyer et al., 2021). The code used for these analyses can be downloaded here: https://osf.io/x92ds/.

## 3 Results

### 3.1 Robustness, precision, accuracy

Gaze data quality analysis showed stable and sufficiently good quality over the course of the testing sessions (see Table 1). More particularly, the high accuracy allowed the discrimination between the gaze directed to the different facial features during affect modulation.

**Table 1:** Eye-tracking robustness, precision and accuracy determined within three validation sequences averaged over all target locations (wall, pre- & post-interaction).

	**Wall**		**Face (pre)**		**Face (post)**	
	**Mean**	**SD**	**Mean**	**SD**	**Mean**	**SD**
**Robustness**						
% valid data	98.25	3.56	95.53	7.16	93.26	12.08
**Precision (SD)**						
In degrees	0.32	0.09	0.44	0.17	0.44	0.18
In cm	0.73	0.21	1.01	0.39	1.01	0.41
**Accuracy**						
In degrees	0.38	0.14	0.52	0.20	0.52	0.26
In cm	0.87	0.32	1.19	0.46	1.19	0.59

The data of quality indices published here have already been used for the evaluation of the used eye-tracking setup and can be found here: Vehlen et al. (2021) (Study 3).

Values in percent, degrees of visual angle and cm. Lower values reflect better precision and accuracy.

### 3.2 Validation of facial affect modulation

Mean intensity of AUs 04, 06 and 12 were used to validate the facial affect modulation—see Figure 1B. As expected, differences in AU04 activation emerged between affect types [*M* ± *SD*; Negative: 1.27 ± 0.35; Positive: 0.07 ± 0.13; *F*_(1, 34)_ = 344.71, *p* < 0.001, $\eta_{\text{p}}^{2}$ = 0.91]. The same was true for activation of AU06 [*M* ± *SD*; Negative: 0.43 ± 0.46; Positive: 1.40 ± 0.20; *F*_(1, 34)_ = 145.52, *p* < 0.001, $\eta_{\text{p}}^{2}$ = 0.81], as well as AU12 [*M* ± *SD*; Negative: 0.29 ± 0.37; Positive: 2.30 ± 0.38; *F*_(1, 34)_ = 834.07, *p* < 0.001, $\eta_{\text{p}}^{2}$ = 0.96]. Furthermore, the time course analysis of AU activation during the 9 s of analysis (Figure 1A) confirmed that peak activation occurred during the 3 s of affect modulation, with moderate variance in the onset timing of these expressions (see Figure 1C). This indicates that the RAs consistently produced the intended facial affect modulation during the required period.

### 3.3 Aggregated measures of gaze behavior

The amount of missing gaze data (due to blinks, excessive head movements, viewing outside the tracking area, etc.) differed between affect types, with more missing values for positive compared to negative affect (*M* ± *SD*; Positive: 38.20% ± 29.00%; Negative: 32.70% ± 27.10%; *t*_(410)_ = 3.31, *p* = 0.001, *r* = 0.16). All subsequent analyses were reported as total dwell times for specific AOIs relative to the total amount of valid data, i.e., total trial length minus missing data.

The first repeated-measures ANOVA (AOI × time × affect type) revealed only a significant main effect of AOIs, *F*_(3.14, 106.92)_ = 19.29, ε = 0.79, *p* < 0.001, $\eta_{\text{p}}^{2}$ = 0.36, with the eyes attracting the most attention on the face of the interaction partner (*M* ± *SD;* Eyes = 37.90 ± 28.40% vs. nose = 12.2 ± 18.20%, mouth = 6.81 ± 13.70% and rest of face = 17.70 ± 19.20%). The main effect of time, *F*_(1.73, 58.66)_ = 0.63, ε = 0.86, *p* = 0.514, $\eta_{\text{p}}^{2}$ = 0.02 and affect type, *F*_(1, 34)_ = 0.66, *p* = 0.422, $\eta_{\text{p}}^{2}$ = 0.02 did not reach significance. Nor did any interaction effect [AOI × affect type: *F*_(1.93, 65.61)_ = 1.94, ε = 0.48, *p* =0.153, $\eta_{\text{p}}^{2}$ = 0.05; AOI × time: *F*_(3.65, 124.23)_ = 1.77, ε = 0.46, *p* =0.146, $\eta_{\text{p}}^{2}$ = 0.05; time × affect type: *F*_(1.98, 67.42)_ = 1.20, ε = 0.99, *p* = 0.308, $\eta_{\text{p}}^{2}$ = 0.03 and AOI × time × affect type: *F*_(3.78, 128.44)_ = 1.37, ε = 0.47, *p* = 0.250, $\eta_{\text{p}}^{2}$ = 0.04].

The following repeated-measures ANOVA for the AOI face showed no significant main effect of affect type, *F*_(1, 34)_ = 3.28, *p* = 0.079, $\eta_{\text{p}}^{2}$ = 0.09. Additionally, no main effect of time, *F*_(1.86, 63.41)_ = 2.89, ε = 0.93, *p* = 0.067, $\eta_{\text{p}}^{2}$ = 0.08 and no time × affect type interaction, *F*_(1.93, 65.59)_ = 2.08, ε = 0.96, *p* = 0.135, $\eta_{\text{p}}^{2}$ = 0.06 could be found. Planned comparisons revealed a significant difference in attention to the face between positive and negative affect during the affect modulation (*M* ± *SD;* Negative: 79.20 ± 28.31%*;* Positive: 70.17 ± 32.63%; *p* = 0.021, $\eta_{\text{p}}^{2}$ = 0.15), but not before and after (all *p* > 0.05; see Figure 1D).

The same pattern emerged for the AOI eyes. The repeated-measures ANOVA revealed no significant main effect of affect type, *F*_(1, 34)_ = 1.48, *p* = 0.232, $\eta_{\text{p}}^{2}$ = 0.05. Again, no significant main effect of time, *F*_(1.77, 60.26)_ = 0.87, ε = 0.89, *p* = 0.413, $\eta_{\text{p}}^{2}$ = 0.03 as well as no significant time × affect type interaction were found, *F*_(1.75, 59.46)_ = 2.96, ε = 0.87, *p* = 0.066, $\eta_{\text{p}}^{2}$ = 0.08. As for the whole face, planned comparisons revealed a significant difference between negative and positive affect during the affect modulation (*M* ± *SD*; Negative: 42.24 ± 29.03%; Positive: 35.41 ± 29.00%; *p* = 0.024, $\eta_{\text{p}}^{2}$ = 0.14) and again not for the time before and after modulation (all *p* > 0.05; see Figure 1E).

Furthermore, no significant main effect of affect type emerged for the AOI mouth, *F*_(1, 34)_ = 3.05, *p* = 0.090, $\eta_{\text{p}}^{2}$ = 0.08. On the contrary, a significant main effect of time was found, *F*_(1.88, 63.76)_ = 5.30, ε = 0.94, *p* = 0.009, $\eta_{\text{p}}^{2}$ = 0.14. The interaction between affect type × time did not reach significance, *F*_(1.60, 54.27)_ = 0.99, ε = 0.80, *p* = 0.363, $\eta_{\text{p}}^{2}$ = 0.03. Planned comparisons revealed no differences in dwell times before, during and after positive compared to negative affect modulation (all *p* > 0.05; see Figure 1F).

### 3.4 Dynamic measures of gaze behavior

The SPLOT method was used to compare gaze behavior in response to negative and positive affect over the course of 9 s (3 s before, during and after affect modulation). Specifically, it was used to provide insight into temporal dynamics during the critical period of affect modulation. Thereby, two clusters emerged in the comparison between positive and negative affect for the AOI face and the AOI eyes, respectively. Differences in look probability on the face emerged around 3,358–4,208 ms (cluster *t*-statistic: 293.21, *p* = 0.078, *d* = 0.48) and 5,258–6,266 ms (cluster *t*-statistic: 305.45, *p* = 0.075, *d* = 0.42; see Figure 2B). Early differences in look probability on the AOI eyes occurred around the same time, namely 3,450–4,225 ms (cluster *t*-statistic: 251.13, *p* = 0.085, *d* = 0.45), and later around 4,908–5,492 (cluster *t*-statistic: 179.82, *p* = 0.189, *d* = 0.43; see Figure 2D). Differences between conditions for the look probability on the AOI mouth during the affect modulation did not occur (see Figure 2F). Although these differences did not reach statistical significance, they indicate attention-related trends in gaze behavior. Specifically, more attention appeared to be directed to the face, particularly the eyes, when the affect of the interaction partner was negative. Conversely, attention to these regions seemed to be reduced during positive affect. These trends align with our hypothesis that negative affect would result in increased attention to the eyes. However, contrary to our hypothesis, positive affect did not lead to greater attention to the mouth region.

## 4 Discussion

The study explored how facial affects influence gaze behavior in natural social interactions. Participants' gaze was recorded using remote eye-tracking during interactions with a RA, and responses to trained affect modulations were observed. A novel method using cluster-based permutation testing captured gaze dynamics alongside aggregated measures.

Facial affect modulations were observed during natural social interactions, which offered fewer opportunities for standardization (Hadders-Algra, 2022; Valtakari et al., 2021). To address the lack of a priori, independent validation of facial expressions, RAs were trained beforehand, and responses were scripted to ensure interaction standardization. Sequences of facial affect modulation were validated using an independent software that provides information about the activation of certain AUs (OpenFace; Amos et al., 2016). As hoped for, positive affect activated AUs linked to happiness (AU06 and AU12), while negative affect primarily triggered an AU associated with anger (AU04). The affect modulation was not subject to strong temporal fluctuations. However, despite RA's precise instructions, we avoided categorizing facial expressions beyond positive and negative.

As expected, the analysis of the aggregated measures revealed a general preference for the eye region over other facial AOIs for all affect types and over time. This highlights the eye region's significance as the most socially salient part of the face in social interactions (Birmingham et al., 2009; Devue et al., 2012; Tatler et al., 2010). Differences in missing values, which can be attributed to noise, blinking, or looking outside the tracking area, were observed across different affect types. Distinguishing between these events could clarify whether positive affect prompts more gaze aversion in this interaction, but an accurate video-based eye-tracking method is yet to be identified (Wisiecka et al., 2022).

In aggregated analyses, subtle differences emerged for the face, specifically the eyes, during negative affect modulation, aligning with increased eye gaze in standardized setups (Calvo et al., 2018). No significant attentional differences occurred for the AOI mouth, contradicting past studies on positive affect (Beaudry et al., 2014; Calvo et al., 2018; Green et al., 2003). However, analyzing only aggregate measurements can result in a loss of information within the high-frequency gaze signal (Belopolsky, 2020).

The SPLOT method maintains temporal resolution without raising the risk of false positives. While not achieving statistical significance, intriguing patterns emerged in participants' gaze behavior during affect modulation. Clusters of rapid changes in both positive and negative affect were identified likely associated with the first saccade in response to affect modulation. Our hypothesis that negative affect would increase attention to the eyes was partially supported by these patterns. Negative affect appeared to direct the gaze toward the face, specifically the eye region. However, contrary to our hypothesis, positive affect did not lead to greater attention to the mouth region, and instead, gaze appeared to be directed away from the face. A similar pattern at the end of the interaction suggested delayed disengagement for negative affect (Belopolsky et al., 2011). Findings regarding negative affect are consistent with those of a more standardized setup using dynamic stimuli (Calvo et al., 2018). Here, angry as well as sad stimuli drew more attention to the eye region. Simultaneously, this change in attention allocation contradicts results of studies using static stimuli (Beaudry et al., 2014; Hunnius et al., 2011). One explanation for these partially consistent results could be the importance of dynamic aspects in the study of facial affect, which is neglected in static stimuli (Krumhuber et al., 2013). Notably, heightened attention to negative affect could stem from its unpredictability in a positive interaction, aligning with research on stimulus unpredictability effects (Becker et al., 2007; Król and Król, 2017). In this context, positive affect could have been subtly noted, possibly explaining the contradictory gaze response. Moreover, participants, engaged in speaking during affect modulation and averted gaze could have enhanced cognitive resources for the heightened demands of communication (*cognitive load theory*; Doherty-Sneddon and Phelps, 2005; Glenberg et al., 1998).

Overall, however, the differences determined using the SPLOT method did not reach significance. Accordingly, the differences in gaze behavior in response to affect modulation were either too small or too brief. This again calls into question the validity of standardized setups in predicting natural gaze behavior (Hietanen et al., 2020; Laidlaw et al., 2011; Risko et al., 2012, 2016). The effects of the facial affect on gaze behavior from more standardized setups could not be clearly replicated in our interactive eye-tracking study. This is in line with a recent study showing no effect of facial affect in a similar interactive setup (Pasqualette and Kulke, 2023). Besides the interaction potential, the influence of task on gaze behavior should be considered when interpreting the results (Hadders-Algra, 2022). While previous studies with a standardized experimental setup often presented the participants with a clear task (rate emotion, arousal, gaze direction, etc.), it can be assumed that natural interactions present evolving demands, which might effect gaze behavior. Additionally, the intensity and length of the gaze response to negative affect could also be attributed to the task, that was developed to generate interpersonal closeness between interaction partners (Aron et al., 1997) and during which negative affect might have been overlooked. However, questions included in the task (e.g., “If you could change one thing about the way you grew up, what would it be? Why?”) can stimulate controversial discussions and potentially results in a negative affective response. Contextual factors may also have influenced the interpretation of emotional facial expressions. Although the questions were presented in a fixed order to maintain consistency, the content of the questions could interact with the displayed facial affect in ways that were not controlled for. For example, an angry facial expression may be perceived differently depending on whether the participant is discussing a neutral topic or a personal grievance. Although the presentation of facial affect was randomized to mitigate order effects, future studies should consider further controlling the context in which emotional expressions are presented.

Another explanation for the absence of significant differences could be the reduced statistical power resulting from a relatively small sample size. Determining the appropriate sample size was challenging because of the considerable variability in effect sizes reported for the interaction effect in previous studies (e.g. AOI × affect: $\eta_{\text{p}}^{2}$ = 0.09; Eisenbarth and Alpers, 2011, or $\eta_{\text{p}}^{2}$ = 0.48; Calvo et al., 2018) and the lack of meta-analyses. In addition, it was difficult to estimate the influence of the naturalistic setting on the anticipated effects. Furthermore, we wanted to look at the data dynamically; to our knowledge, no previous studies have observed the effects of affect over time. This also made it difficult to predict the exact sample size, as gaze behavior is expected to become significantly noisier over time. Combined with a complex study design that limited the feasible sample size, we decided to address threats to statistical power through increasing measurement precision (Hedge et al., 2018; Nebe et al., 2023; Smith and Little, 2018). This involved excluding participants with compromised gaze data quality, using a validated eye-tracking setup (Tönsing et al., 2022; Vehlen et al., 2021), assessing data quality twice (pre- and post-interaction), and validating facial affect modulation.

Apart from the above limitations, the present data, together with several other recent studies, suggest potential differences in gaze behavior during social interactions compared with gaze behavior in more standardized setups. Brief facial affect modulation within positive social interactions appears to have short-term rather than fundamental effects on healthy participants' gaze behavior. To validate these preliminary findings and enhance their generalizability, subsequent studies should replicate this research with larger sample sizes. Moreover, future research should explore gaze behavior across diverse social interactions, encompassing a broader range of facial expressions. Furthermore, the additional analysis of the interaction partners' gaze behavior can illuminate the interplay of these signals in the interactional context. Together with information from other modalities, such as the analysis of speech, this can ultimately enable a deeper understanding of dynamic systems such as social interactions. Consequently, these findings offer valuable initial insights into the impact of affect on gaze behavior, providing a foundation for future inquiries into effect sizes, analytical approaches, and measurement precision.

## Data Availability

The datasets presented in this study can be found in online repositories. The names of the repository/repositories and accession number(s) can be found at: https://osf.io/x92ds/.
