# Supplementary material for: Gaze behavior in response to affect during natural social interactions
Source: Front Psychol. 2024 Oct 14;15:1433483. doi: 10.3389/fpsyg.2024.1433483 (PMC11514276; doi:10.3389/fpsyg.2024.1433483)
Supplement: Supplementary file 1 [file Data_Sheet_1.docx]

Supplementary Material

# Supplementary Information on the face-to-face interaction

The face-to-face interaction sequences were structured by the following 12 questions adapted from an evaluated and published paradigm (Aron et al., 1997):

1. Given the choice of anyone in the world, whom would you want as a dinner guest?

2. Would you like to be famous? In what way?

3. Before making a telephone call, do you ever rehearse what you are going to say? Why?

4. What would constitute a “perfect” day for you?

5. When did you last sing to yourself? To someone else?

6. If you were able to keep either the mind or the body of a 25-year-old for your entire life, which would you choose? Why?

7. Name three things you and your partner appear to have in common.

8. If you could wake up tomorrow having gained any one quality or ability, what would it be? Why exactly this one?

9. For what in your life do you feel most grateful? Why?

10. If you could change anything about the way you were raised, what would it be? Why?

11. If a crystal ball could tell you the truth about yourself, your life, the future, or anything else, what would you want to know? Why?

12. Take 3 minutes and tell your partner your life story.

**Table S1**

*Detailed information on collected psychometric measures*

| Measure | *N* | Mean | *SD* | Range | Percentage above clinical cut-off |
| --- | --- | --- | --- | --- | --- |
| Autism-Quotient (AQ-k) | 35 | 8.94 | 4.55 | 3-19 | 5.71 %  (≥ 17) |
| Social Interaction Anxiety Scale (SIAS) | 35 | 23.91 | 11.54 | 5-43 | 34.29 % (> 30) |
| Gaze Anxiety Rating Scale (GARS) | 35 | 23.66 | 11.76 | 2-49 | - |
| Brief Fear of Negative Evaluation (FNE-k) | 35 | 35.86 | 9.90 | 17-58 | - |

*Notes*. *Autism Quotient – German short version* (AQ-k; Freitag et al., 2007), *Brief Fear of Negative Evaluation – German short version* (FNE-k; Reichenberger et al., 2016), *Gaze Anxiety Rating Scale – German version* (GARS; Domes et al., 2016), *Social Interaction Anxiety Scale – German version* (SIAS; Stangier et al., 1999). Clinical cut-offs indicated in parentheses. No clinical cut-offs available for German versions of GARS and FNE-k.

**References**

Aron, A., Melinat, E., Aron, E. N., Vallon, R. D., & Bator, R. J. (1997). The experimental generation of interpersonal closeness: A procedure and some preliminary findings. *Personality and Social Psychology Bulletin*, *23*(4), 363–377. https://doi.org/10.1177/0146167297234003

Domes, G., Marx, L., Spenthof, I., & Heinrichs, M. (2016). The German version of the Gaze Anxiety Rating Scale (GARS): Reliability and validity. *PloS One*, *11*(3), e0150807. https://doi.org/10.1371/journal.pone.0150807

Freitag, C. M., Retz-Junginger, P., Retz, W., Seitz, C., Palmason, H., Meyer, J., Rösler, M., & von Gontard, A. (2007). Evaluation der deutschen Version des Autismus-Spektrum-Quotienten (AQ)—Die Kurzversion AQ-k. *Zeitschrift für Klinische Psychologie und Psychotherapie*, *36*(4), 280–289. https://doi.org/10.1026/1616-3443.36.4.280

Reichenberger, J., Schwarz, M., König, D., Wilhelm, F. H., Voderholzer, U., Hillert, A., & Blechert, J. (2016). Angst vor negativer sozialer Bewertung: Übersetzung und Validierung der Furcht vor negativer Evaluation–Kurzskala (FNE-K). *Diagnostica*, *62*(3), 169–181. https://doi.org/10.1026/0012-1924/a000148

Stangier, U., Heidenreich, T., Berardi, A., Golbs, U., & Hoyer, J. (1999). Die Erfassung sozialer Phobie durch Social Interaction Anxiety Scale (SIAS) und die Social Phobia Scale (SPS). *Zeitschrift Für Klinische Psychologie*, *28*(1), 28–36. https://doi.org/10.1026//0084-5345.28.1.28
